# How important is thermodynamics for identifying elementary flux modes?

**DOI:** 10.1371/journal.pone.0171440

**Published:** 2017-02-21

**Authors:** Sabine Peres, Mario Jolicœur, Cécile Moulin, Philippe Dague, Stefan Schuster

**Affiliations:** 1 LRI, Université Paris-Sud, CNRS, Université Paris-Saclay, 91405 Orsay, France; 2 MaIAGE, INRA, Université Paris-Saclay, 78350 Jouy-en-Josas, France; 3 Research Laboratory in Applied Metabolic Engineering, Department of Chemical Engineering, École Polytechnique de Montréal, P.O. Box 6079, Centre-ville Station, Montreal (Quebec), Canada; 4 Department of Bioinformatics, Friedrich Schiller University of Jena, 07743 Jena, Germany; Tata Institute of Fundamental Research, INDIA

## Abstract

We present a method for computing thermodynamically feasible elementary flux modes (tEFMs) using equilibrium constants without need of internal metabolite concentrations. The method is compared with the method based on a binary distinction between reversible and irreversible reactions. When all reactions are reversible, adding the constraints based on equilibrium constants reduces the number of elementary flux modes (EFMs) by a factor of two. Declaring in advance some reactions as irreversible, based on reliable biochemical expertise, can in general reduce the number of EFMs by a greater factor. But, even in this case, computing tEFMs can rule out some EFMs which are biochemically irrelevant. We applied our method to two published models described with binary distinction: the monosaccharide metabolism and the central carbon metabolism of Chinese hamster ovary cells. The results show that the binary distinction is in good agreement with biochemical observations. Moreover, the suppression of the EFMs that are not consistent with the equilibrium constants appears to be biologically relevant.

## 1 Introduction

The notion of elementary flux mode (EFM) is a key concept in the analysis of metabolic networks from a pathway-oriented perspective. An EFM is defined as a smallest (with respect to reactions set inclusion) sub-network that enables the metabolic system to operate at steady state with all irreversible reactions proceeding in the appropriate direction [1, 2]. The idea traces back to the concept of extreme currents proposed by Clarke [3]. EFMs analysis has been used successfully in analyzing multitudinous biochemical networks including fatty acid synthesis in plant seeds [4], recombinant protein synthesis in *Escherichia coli* [5] and penicillin synthesis [6]. The main drawback of this approach is the huge number of EFMs associated with large biochemical networks which prevents from drawing simple conclusions from their analysis. That drawback is severe in view of the genome-scale metabolic reconstruction for an ever increasing number of organisms [7, 8]. The set of EFMs represents the potential pathways in a metabolic network but the biologically active pathways are limited by various biological constraints: thermodynamic constraints, kinetics and regulations. Acuña *et al*. [9, 10] gave a systematic overview of the complexity of the optimisation problems related to EFMs and showed that counting EFMs is #P-complete (which is a special complexity class related to counting problems associated with decision problems in the class NP). Being a computationally demanding task, several approaches to parallel or distributed computation of EFMs have been proposed through parallelization techniques [11] or algorithmic reformulations [12–14]. In the traditional method for calculating EFMs, a binary distinction is made between reversible and irreversible reactions. Although this distinction is sometimes not easy to make, it is known for many biochemical reactions whether they are reversible or irreversible. This information is given in original papers, in textbooks, on pathway charts such as the Boehringer map and in databases such as KEGG [15]. However, this binary distinction is an oversimplification because, in principle, any reaction is reversible. This can be explained by an argument based on the second law of thermodynamics. In a strictly irreversible reaction, the concentration(s) of substrate(s) would tend to zero as time proceeds. This, however, would imply the unrealistic case that entropy would diverge because it is a function of the logarithm of concentrations [16]. In reality, there are quantitative differences according to the equilibrium constants rather than a qualitative difference between reversible and irreversible. If the equilibrium constant is very high (or much lower than unity), the reaction can be considered practically irreversible in the forward direction (respectively backward direction). As EFM analysis has been applied successfully for about 20 years, the above-mentioned oversimplification cannot be a major problem. Nevertheless, the question arises on what error is made by the binary distinction between reversible and irreversible reactions. It has been suggested repeatedly that thermodynamic information, notably the equilibrium constants, should be included in the analysis because this would be a more precise approach. The number of applications of thermodynamics-based network analysis methods have been increasing in the last ten years [17]. The main important use of thermodynamics-based analysis is the determination of reaction directionality, whereby the feasibility of reaction fluxes or flux distributions can be checked based on calculation of changes in Gibbs free energy using metabolite concentrations. Hoppe et al. [18, 19] proposed an algorithm that takes into account the metabolite concentrations to assign flux directions in flux balance analyses. Kümmel *et al*. [20] developed an algorithm based on thermodynamics and network topology to assign the direction of the reactions with respect to the production of energy equivalents. The energy balance analysis (EBA), defined by Beard *et al*. [21], constrains the bounds of the fluxes using the Gibbs free energy. The authors then defined sign patterns of fluxes and analysed which of them are thermodynamically feasible [22]. They did so by analysing the internal cycles space and using the theory of oriented matroids. Internal cycles do not perform a net transformation of external metabolites and should, thus, be excluded from the set of relevant EFMs. Henry *et al*. [23] presented the thermodynamics-based flux analysis which uses mixed-integer linear programming formulation and computes the flux directionality based on the thermodynamically feasible concentration profiles. Jol *et al*. [24] characterized the flux solution space by determining EFMs that are subsequently classified as thermodynamically feasible or infeasible on the basis of experimental metabolome data. They incorporated quantitative metabolite concentrations into the analysis of the space of all stoichiometrically feasible flux distributions. They first used flux variability analysis (FVA) [25] to determine reversibility of each reaction. Then, they used network-embedded thermodynamic analysis (NET analysis) [26] to determine additional reaction direction constraints based on the activities of reactions that are active for all flux distributions. They computed all EFMs and used quantitative metabolite data to test the activities of each EFM for thermodynamic feasibility. Müller *et al*. [27] presented direct method for computing modules of the thermodynamically constrained optimal flux space of a metabolic network. This method can be used to decompose the set of optimal-yield elementary flux modes in a modular way and to speed up their computation. More recently, Gerstl *et al*. [28–30] integrated linear programming in efmTOOL [14] to compute thermodynamically feasible EFMs during the enumeration process. Most of the above-mentioned methods are based on thermodynamics and use metabolome data, that is, concentration values of metabolites. However, such data in many cases are not available. In contrast, the equilibrium constants are relatively easy to obtain because they are independent of the properties of the enzymes. These constants can be found in a very useful database eQuilibrator [31] which used methods developed by Noor *et al*. [32, 33] based on thermochemical estimation. Moreover, several approaches have been published for computing equilibrium constants (or the equivalent standard Gibbs free energy differences) from properties of the molecules involved [34–36]. Admittedly, the concentrations of external metabolites (or equivalent information) are needed in addition to equilibrium constants for a thermodynamic description because they determine the distance from equilibrium and affect the directionality of reactions. But, even if they are more difficult to obtain than equilibrium constants, they are often easier to acquire than the concentrations of internal metabolites. In this paper, we analyze what error is made in the traditional approach based on a binary distinction between reactions. In particular, we analyze whether EFMs are computed that are not biologically relevant in reality and whether some relevant EFMs are not computed. And we compare, from the point of view of efficiency and accuracy in the computation of relevant EFMs, this approach, which uses given biochemical-based knowledge about irreversibility of some reactions, to the approach that uses more quantitative thermodynamic information. To this end, we derive a method for predicting the set of relevant EFMs, that is, the modes consistent with thermodynamic constraints without need of internal metabolite concentrations. In our analysis, we use a Lemma proved earlier [37] and illustrate the comparison by several examples.

## 2 Results

### 2.1 Thermodynamics versus irreversibility biochemical knowledge for computing EFMs

In this section, we consider the case that all reactions in a system are, in principle, reversible and we do not know for all of them at the beginning in which direction they will operate at steady state. One way of finding out the directionality is to compute all EFMs for the case that all reactions in the system are reversible and check for each EFM, by using the inequality Eq (20) shown in section 3.1, whether it can proceed in the forward direction. If the inequality Eq (20) is fulfilled with the opposite order relation, the EFM operates in the reverse direction.

#### Case of a linear chain

To illustrate this, consider the case of a linear chain of *n* monomolecular reactions, oriented positively from external metabolite $M_{1}={\bar{X}}_{1}$ to external metabolite $M_{n+1}={\bar{X}}_{2}$. We saw that the necessary thermodynamic feasibility condition is given by inequality Eq (14), which is nothing else that an instance of the general condition Eq (20) for the EFM given by the chain (represented by the unit vector). It can be rewritten as:

$$\begin{array}{c} \frac{{\bar{X}}_{2}}{{\bar{X}}_{1}}<\prod_{j=1}^{n}K_{j} \end{array}$$ (1)

Now, suppose for the moment that we have not yet considered the steady-state assumption and that we know nothing about the direction in which each of the *n* reactions proceeds. This means that each reaction may proceed either forward or backward. So, there are theoretically 2^n^ possible configurations. Keeping the convention to define forward direction for all reactions from ${\bar{X}}_{1}$ to ${\bar{X}}_{2}$, changing the direction of reaction *j* boils down to replacing in Eq (1) *K*_*j*_ with ${K_{j}}^{-1}$ (and also ${\bar{X}}_{1}$ with ${{\bar{X}}_{1}}^{-1}$ for *j* = 1, and ${\bar{X}}_{2}$ with ${{\bar{X}}_{2}}^{-1}$ for *j* = *n* − 1). Let us define:

$$\begin{array}{c} K=min_{\lambda_{j}\in \left\{K_{j},{K_{j}}^{-1}\right\}}\prod_{j=2}^{n-1}\lambda_{j} \end{array}$$ (2)

We have 0 < *K* ≤ 1 and, at least theoretically, we see that, if the concentrations of external metabolites are imposed such that $\frac{{\bar{X}}_{2}}{{\bar{X}}_{1}}<K_{1}K_{n}K$, then the thermodynamical feasibility condition Eq (1) is satisfied for reactions 1 and *n* in forward direction, whatever the direction of any of the *n* − 2 other reactions *j*, 2 ≤ *j* ≤ *n* − 1. In the same way, if ${\bar{X}}_{2}{\bar{X}}_{1}<{K_{1}}^{-1}K_{n}K$, Eq (1) is satisfied for reaction 1 in backward direction and reaction *n* in forward direction, whatever the direction of any of the *n* − 2 other reactions. This means that choosing for example ${\bar{X}}_{1}=1$ and ${\bar{X}}_{2}<min\left(K_{1},{K_{1}}^{-1}\right)K_{n}K$ satisfies Eq (1) for reaction *n* in forward direction, whatever the direction of any of the *n* − 1 other reactions. Conversely, the same reasoning shows that choosing ${\bar{X}}_{1}=1$ and ${\bar{X}}_{2}>min^{-1}\left(K_{1},{K_{1}}^{-1}\right)K_{n}K^{-1}$ satisfies Eq (1) for reaction *n* in backward direction, whatever the direction of any of the *n* − 1 other reactions. So, with ${\bar{X}}_{1}=1$ and depending on the value of ${\bar{X}}_{2}$, either smaller than $min\left(K_{1},{K_{1}}^{-1}\right)K_{n}K$ or greater than $min^{-1}\left(K_{1},{K_{1}}^{-1}\right)K_{n}K^{-1}$ (both conditions are obviously exclusive as $min(K_{1},{K_{1}}^{-1})\leq 1$ and *K* ≤ 1), then necessary thermodynamical feasibility condition will just fix that reaction *n* proceeds either in forward or backward direction, all the *n* − 1 other reactions being able to proceed independently in any direction (an equivalent reasoning can be done by exchanging ${\bar{X}}_{1}$ and ${\bar{X}}_{2}$, and reactions 1 and *n*). This means that thermodynamical feasibility condition just divides by two the configuration space size, from 2^*n*^ to 2^*n* − 1^. By comparison, the steady-state condition Eq (15) imposes that every internal metabolite that is produced has to be consumed and thus imposes that all *n* reactions have to proceed in the same direction, leaving only two configurations (forward or backward pathway), and thus two possible exclusive EFMs, and thus dividing by 2^*n* − 1^ the configuration space size, from 2^*n*^ to 2. Adding the thermodynamical feasibility condition to the steady-state condition provides a unique configuration, either forward or backward depending on Eq (1) being satisfied or not, and thus a unique EFM. The same result can be actually obtained by adding a reliable irreversibility condition for an arbitrary reaction among the *n* ones.

#### Case of reaction chains connecting to a hub metabolite

In a more general case, consider now linear chains of monomolecular reactions sharing exactly one internal “hub” metabolite *X*. So, suppose *k* linear chains of size *p*_*k*_ from external metabolites ${\bar{X}}_{k}$ to internal metabolite *X* that are independent (except for *X*, the unique common metabolite). This network has $\sum_{i=1}^{k}p_{i}$ reactions, $\sum_{i=1}^{k}p_{i}-k+1$ internal metabolites and *k* external metabolites. There are $\frac{k(k-1)}{2}$ possible distinct EFMs supports (i.e., independently of the directions of the reactions), obtained by all choices of two external metabolites among the *k* ones. In absence of knowledge about the direction of each reaction, the configuration space has size $2^{\sum_{i=1}^{k}p_{i}}$.

From the reasoning above on a linear chain, we can conclude that for an arbitrary (forward or backward) direction imposed on each of the *k* − 1 reactions involving ${\bar{X}}_{i}$ with 2 ≤ *i* ≤ *k*, there exists a choice of the concentrations of external metabolites such that we obtain $2^{\sum_{i=1}^{k}p_{i}-k+1}$ thermodynamically feasible configurations. Thus, thermodynamics in the absence of knowledge of the concentrations of internal metabolites may divide the configuration space size only by 2^*k* − 1^.

Now, applying the steady-state assumption to the initial configuration space imposes the same direction (forward or backward) on all reactions in each of the *k* chains connected to *X*. Moreover, the stationarity of concentration *X* suppresses two impossible configurations. This leads to 2^*k*^ − 2 configurations. This means that the steady-state assumption alone divides the configuration space size approximately by $2^{\sum_{i=1}^{k}\left(p_{i}-1\right)}$, much more than thermodynamics alone.

Now, imposing both the steady-state assumption and thermodynamic feasibility gives a configuration space of size *k* − 1. Configurations are given by the *i* first chains directed from their external metabolite towards *X* and the *k* − *i* last ones directed from *X* to their external metabolite, for 1 ≤ *i* ≤ *k* − 1. Thus we obtain *i*(*k* − *i*) EFMs. Instead of using thermodynamics, adding a reliable irreversibility condition for an arbitrary reaction in each of the *k* linear chains would obviously lead to a single one configuration. If we are interested only in the EFMs, steady-state assumption gives rise to *k*(*k* − 1) possible EFMs organized into 2^*k*^ − 2 possible exclusive sets of simultaneously consistent EFMs, whose number varies between *k* − 1 and $\left\lfloor \frac{k^{2}}{4}\right\rfloor$. Again, using in addition thermodynamics divides by two the number of EFMs, which is thus equal to $\frac{k(k-1)}{2}$. Knowing instead the direction of k reactions belonging to different chains would divide the number of EFMs by at least 4 and at most k, so a better result than with thermodynamics.

#### Example with four reactions connecting to a hub metabolite

**Fig 1:**
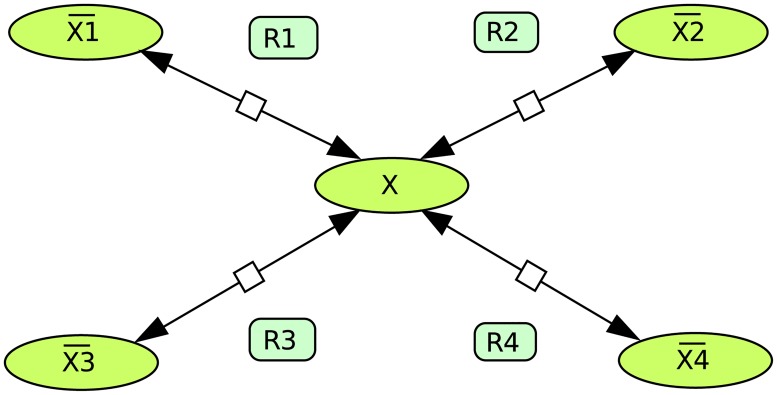
Simple metabolic network example.

**Fig 2:**
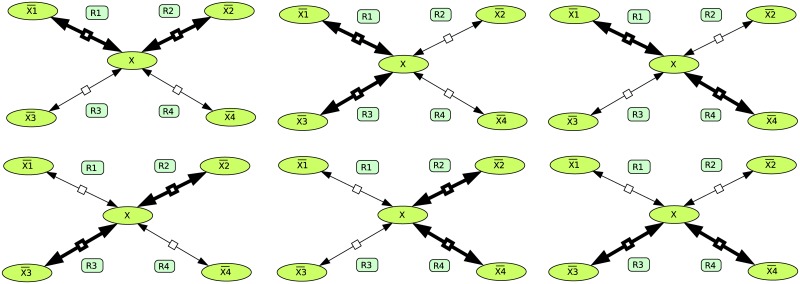
Reversible EFMs of the simple metabolic network example.

Now let us consider the network shown in Fig 1. The thermodynamic information is given by the equilibrium constants *K*_1_, *K*_2_, *K*_3_ and *K*_4_ and the concentrations of the four external metabolites ${\bar{X}}_{i}$. Now we assume (perhaps hypothetically) that all reactions are reversible even if the thermodynamic information determines irreversibility for some reactions already (we fix arbitrarily the forward direction of the four reactions from left to right in the figure). Now we compute the EFMs, giving the 6 reversible EFMs: (1 1 0 0), (1 0 −1 0), (1 0 0 1), (0 1 1 0), (0 −1 0 1), (0 0 1 1) shown in Fig 2. Let us, for example, assume that the Gibbs free energy differences have the following signs:

$$\begin{array}{c} \Delta G_{2,1}<0, \end{array}$$ (3a)

$$\begin{array}{c} \Delta G_{3,2}<0, \end{array}$$ (3b)

$$\begin{array}{c} \Delta G_{4,3}<0. \end{array}$$ (3c)

where the indices *i*, *j* mean that the difference between the energies of the *i* − *th* and *j* − *th* metabolites are taken. This means that the Gibbs free energies of ${\bar{X}}_{1}$, ${\bar{X}}_{2}$, ${\bar{X}}_{3}$ and ${\bar{X}}_{4}$ are descending in this order. On the basis of this thermodynamic information, we check in which direction each EFM can operate. The EFM (1 1 0 0) can operate in the forward direction because relation Eq (20) reads in this case

$$\begin{array}{c} \log{\hat{K}}_{1}+\log{\hat{K}}_{2}>0 \end{array}$$ (4)

which is equivalent to Eq (3a). The EFM (1 0 −1 0), can operate in the forward direction as well because Eqs (3a, 3b) imply Δ*G*_3,1_ < 0. In contrast, the EFM (0 1 1 0) needs to be reverted into (0 −1 −1 0) because of Eq (3b). The remaining three EFMs can operate in the forward direction.

Now, we compare this result with the result we would obtain if we made a binary distinction between reversible and irreversible reactions and did not include quantitative thermodynamic information. However, some qualitative thermodynamic information is necessary to make the binary distinction. It may be known from biochemical experiments that there is a steep thermodynamic gradient between ${\bar{X}}_{1}$ and ${\bar{X}}_{4}$. Then it is sensible to define reactions 1 and 4 as irreversible and the others as reversible. This would give rise to the following 6 EFMs: (1 1 0 0), (1 0 −1 0), (1 0 0 1), (0 1 1 0), (0 −1 0 1), (0 0 1 1). All EFMs except (0 1 1 0) are irreversible. For (0 1 1 0), also the opposite is allowed: (0 −1 −1 0). Now we can see that this uncertainty is the only difference to the result obtained for this example by the thermodynamic approach. All the other EFMs are identical. Only one false positive result is obtained and no false negative.

#### General case

Let us now turn to the general case of reaction networks of higher complexity. These should, however, still be tractable in the sense that all EFMs should be computable for the case where all reactions are considered reversible. Thus, combinatorial explosion should not be too drastic. As an output of the traditional EFM algorithms, all EFMs are reversible under the conditions mentioned. However, for given concentrations of external metabolites, only one direction is feasible for any one EFM under consideration. Therefore, it is of interest to compare the number of such EFMs with the number of EFMs obtained when some reactions are considered irreversible due to biochemical knowledge.

### 2.2 An example network from monosaccharide metabolism

**Fig 3:**
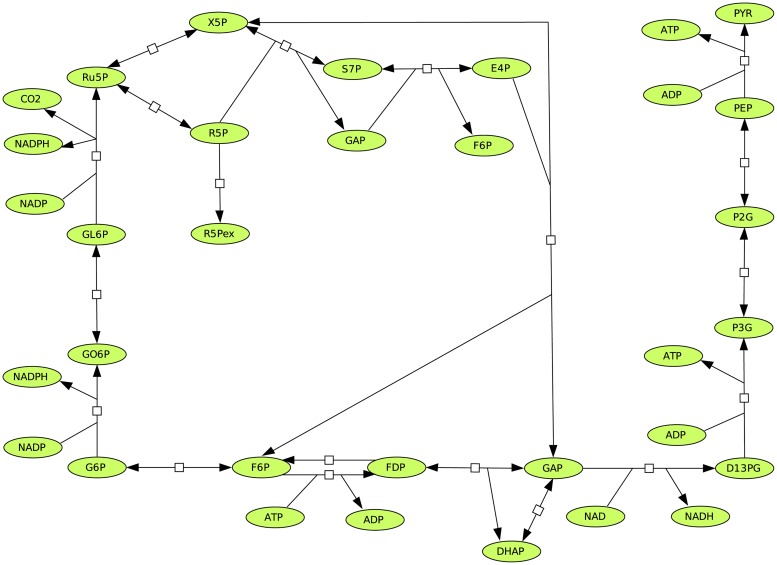
Monosaccharide metabolism.

Let us consider, for example, the system of monosaccharide metabolism (see Fig 3) analyzed earlier [2]. When all reactions are considered reversible, running Metatool (or any other appropriate program) shows that 19 pairs of EFMs are found. In the cited paper, eight reactions such as phosphofructokinase, fructose-biphosphatase and glucose-6-phosphate dehydrogenase are considered irreversible (see Fig 3). This leads to only seven EFMs (EFM 1—EFM 7 in supporting information S1 File) which are all consistent with the thermodynamics. We used the formula Eq (20) to check if the EFMs are thermodynamically feasible (noted tEFMs). In the earlier analysis of the monosaccharide metabolism example [2], the case where ribose-5-phosphate (R5Pex) can not only be consumed but also produced, was considered as well. In that case, the number of EFMs increases to 13. The question arises whether for each of the remaining 6 pairs of modes, one directionality is feasible (EFM 8—EFM 13 in supporting information S1 File). This should be feasible from a thermodynamic point of view, as explained above. However, whether or not an EFM operates does not only depend on thermodynamics but also on kinetics. It may happen that some enzyme is inhibited whenever some other is operating. The biological functions of metabolism may imply that certain routes should be avoided. Moreover, reverting the direction of a reaction might only be feasible if the product concentration gets very high, which is often impossible in living cells. Thus, the decision whether some enzyme is defined to be irreversible is based not only on thermodynamics but also on information on (or biochemical experience with) gene expression, metabolic activation, concentrations values etc. [38].

We have analyzed the 6 additional pairs of EFMs computed in the completely reversible case (EFM 14—EFM 19 in supporting information S1 File). It has turned out that most of tEFMs use either phosphofructokinase or fructose-bisphosphatase in the reverse direction. However, while phosphate donors other than ATP, such as pyrophosphate, are used by phosphofructokinase in some organisms, reverse fructose-bisphosphatase (which would imply inorganic phosphate to be a co-substrate) has never been observed, to the best of our knowledge. It can be assumed that such an operation mode of the enzyme would imply unrealistically high concentrations of fructose-6-phosphate and/or phosphate [38]. In summary, the set of EFMs computed earlier [2] based on the binary distinction is in good agreement with biochemical observations. However, it should be noted that for the EFMs 13, 16 and 19, the conclusion is less clear since the reversibility of some enzymes (e.g. glyceraldehyde-P-dehydrogenase, 3-phosphoglycerate-kinase) is still questioned [39].

### 2.3 Application to central carbon metabolism of Chinese hamster ovary (CHO)

**Fig 4:**
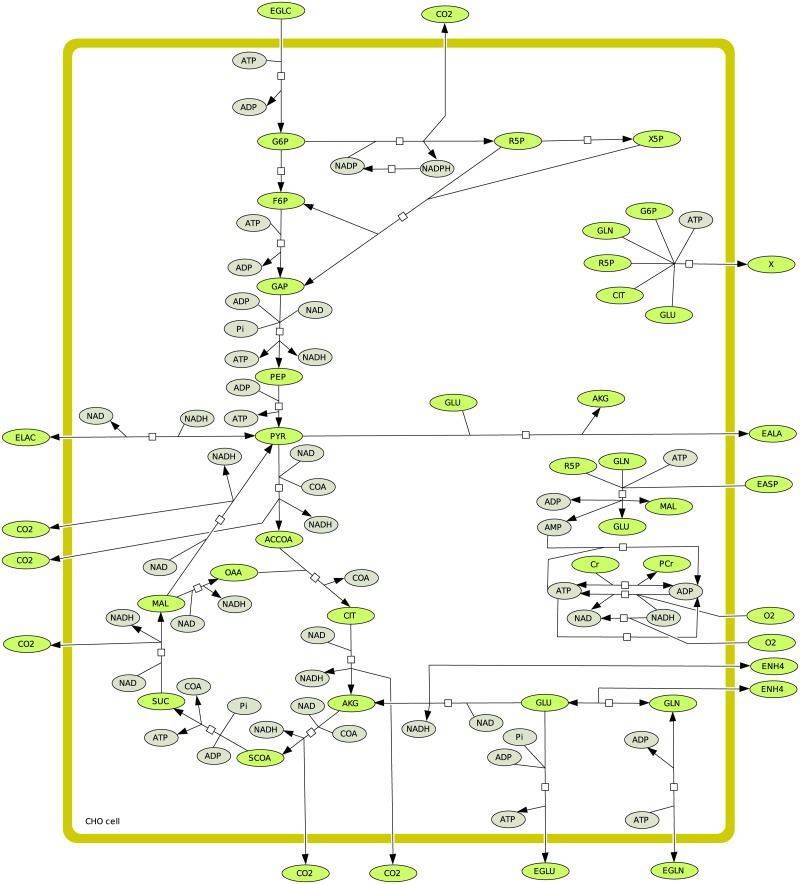
CHO metabolism.

In this section, we analyze the central carbon metabolism of Chinese hamster ovary (CHO) cells, based on a metabolic network that includes glycolysis, the pentose phosphate pathway, TCA cycle, respiratory chain, redox state and energetic metabolism [40]. The metabolic network has been previously validated developing a kinetic-metabolic model describing and simulating the behavior of CHO cells. Cell growth is defined as a function of the major precursors for the synthesis of cell building blocks (G6P, R5P, CIT). Calculations of the EFMs have been performed using experimental data of bioreactor CHO cell cultures previously published [41], and onto which the kinetic-metabolic model has been adapted. Data at *T* = 0*h* correspond to culture conditions at inoculation, while data at *T* = 48*h* correspond to mid-exponential phase, *T* = 72*h* correspond to late exponential phase and *T* = 96*h* represent stationary growth phase. The model has 30 reactions including 25 irreversible reactions and 37 metabolites including 12 external metabolites (see Fig 4). Using Metatool [12], we obtained 31 EFMs. They were drawn using a matlab function EFMdraw that we had implemented and connected to Metatool. All these EFMs are irreversible and all the reactions are used in only one direction.

**Fig 5:**
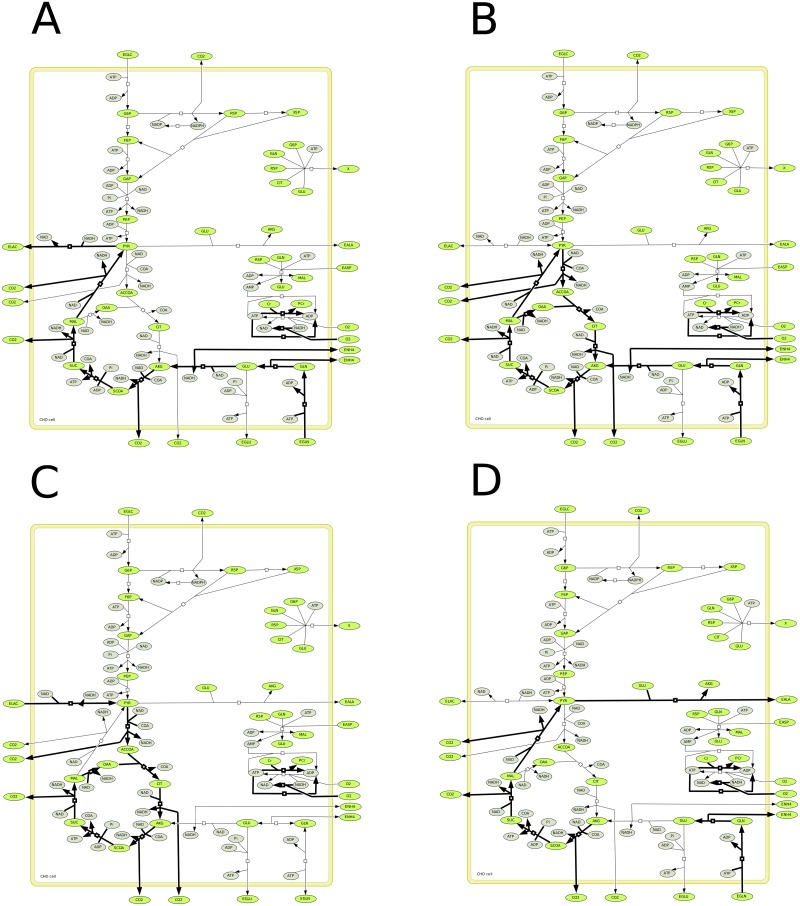
Non feasible EFMs of the CHO model at T = 0.

**Fig 6:**
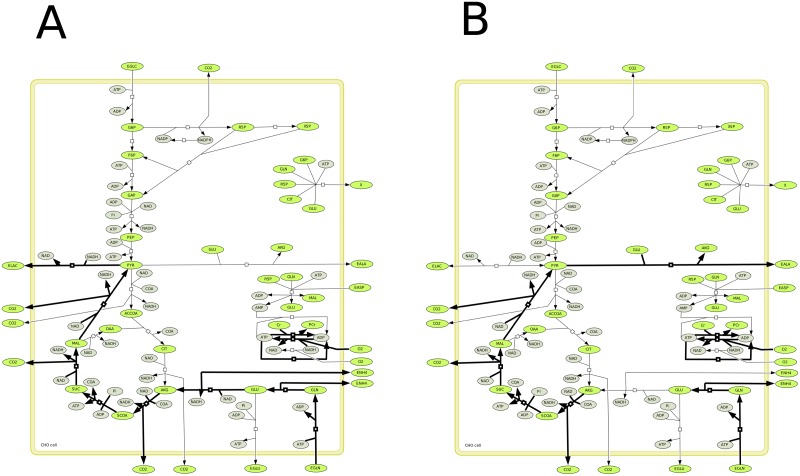
EFMs of the CHO model which become non feasible after T = 48h.

We then verified if the EFMs were consistent with thermodynamics. The model contains 27 tEFMs at *T* = 0 and 26 tEFMs at *T* = 48*h*, *T* = 72*h* and *T* = 96*h*. The non-thermodynamic EFMs at *T* = 0*h* are: [8 16 18 23] (shown in the Fig 5), whereas at *T* = 48, 72, 96*h* they are: [8 13 15 16 23] (shown in Fig 6). EFM 8 is most improbable because it is known that lactate is mostly coming from glucose and not from glutamine [42]. In the model, we only describe one pool of pyruvate (*i*.*e* the cytosolic pyruvate is not differentiated from mitochondrial pyruvate). It is known that lactate comes from cytosolic pyruvate while alanine comes from mitochondrial pyruvate (which arises from glutamine). Moreover, after *T* = 48*h* the concentration of glutamine decreases and is not enough to be assimilated. For the same reason, the EFM 13 and EFM 16 cannot be thermodynamically feasible. At *T* = 0, the cells are entering the exponential growth phase. Therefore, the EFM 16 makes no biological sense with ATP consumption for the transformation of extracellular glutamine into extracellular alanine, while cells massively need supporting anabolic activity using intermediate metabolites as precursors as well as the ATP regenerated. EFM 18 cannot be feasible as well as there is no lactate in the medium but it can be feasible thereafter because the concentration of lactate increases. EFM 23 comes down to CO_2_ production and ATP consumption by ATPase, such as a futile cycle. The thermodynamical infeasibility of this EFM seems pertinent because a major problem of this EFM is that the flux associated to proton leak is amplified compared to biological expected levels, and this flux may thus serve as a fitting parameter reaching improbable level. At time 48h, the cells are in the middle of the exponential growth phase. Therefore, the EFM 15 and EFM 16 are barely possible while cells require precursors and ATP as stated above looking at the EFMs at *T* = 0. More importantly, these pathways can barely be active since glutamine is decreasing from 72h in the culture medium to low level that does not allow the EFM thermodynamical feasibility. It is worth noting that if we suppress the reaction leak because this reaction normally accounts for less than 20 (percent) of *O*_2_ consumption in reality, all the EFMs are thermodynamically feasible at *T* = 0*h*.

Let us now consider all the reactions of the CHO network as reversible. The new network contains 7,529 reversible EFMs and so 7,529 tEFMs since they have all to verify Eq (20) for some orientation, which changes in function of the external concentrations. For example, the number of tEFMs which consume glucose to produce biomass increases from 2,610 at *T* = 0*h* to 2,852 at *T* = 96*h*. The number of tEFMs which consume glucose to produce lactate is on average the same from 1,003 at *T* = 0*h* to 1,013 at *T* = 96*h*. Moreover, for each period of time *T*, we have tested if the thermodynamics imposes some reactions to be irreversible. We verified if there exists reactions that always operate in the same direction in each set of tEFMs. Actually, this is not the case, i.e. all the reactions remain reversible, which means that the knowledge of the equilibrium constants is not enough to replace (even partially) the binary distinction of the expert. And the example of the six non-thermodynamical EFMs above, depending on the context, show that, for biochemical reasons, they are not feasible. And these reasons are not reducible to thermodynamics (otherwise, they should be feasible with the reverse orientation).

## 3 Materials and methods

### 3.1 Thermodynamic description

We denote the concentrations of internal and external metabolites to the system by *X*_*i*_ and ${\bar{X}}_{i}$, respectively. When writing general equations for all metabolites (including internal and external metabolites), we write *M*_*i*_. The stoichiometric coefficients for metabolites *i* in reactions *j* are denoted by *s*_*ij*_ and combined into the *m* × *r* real stoichiometry matrix *S*, where *m* is the number of internal metabolites and *r* the number of reactions. It is known from thermodynamics that the Gibbs free energy difference must be negative in order that a reaction *j* can proceed in the forward direction [16]:

$$\begin{array}{c} \Delta G_{j}=\Delta G_{j}^{0}+RT\ln\prod_{i}M_{i}^{s_{ij}}<0 \end{array}$$ (5)

where $\Delta G_{j}^{0}$ is the standard free energy change. Note that the stoichiometric coefficients for products are positive, while those for substrates are negative. We denote the former by $s_{ij}^{+}$ and the absolute value of the latter by $s_{ij}^{-}$. One obtains the formula for $\Delta G_{j}^{0}$ by considering thermodynamic equilibrium (i.e., Δ*G*_*j*_ = 0). One gets:

$$\begin{array}{c} \Delta G_{j}^{0}=-RT\ln K_{j} \end{array}$$ (6)

with

$$\begin{array}{c} K_{j}=\frac{\prod_{i}{M_{i}^{=}}^{s_{ij}^{+}}}{\prod_{i}{M_{i}^{=}}^{s_{ij}^{-}}} \end{array}$$ (7)

where $M_{i}^{=}$ are the concentrations of metabolites at equilibrium, thus *K*_*j*_ is the equilibrium constant of reaction *j*.

For non-equilibrium states where the reaction *j* proceeds in the forward direction (Δ*G*_*j*_ < 0) we can, using Eq (6), rewrite Eq (5) as:

$$\begin{array}{c} \frac{\prod_{i}M_{i}^{s_{ij}^{+}}}{\prod_{i}M_{i}^{s_{ij}^{-}}}<K_{j} \end{array}$$ (8)

This equation is intuitively understandable: if the concentrations of substrates are high, the reaction proceeds in the forward direction, while it would run backwards if the concentrations of products were sufficiently high.

For those reactions *j* that involve external metabolites, we move their concentrations to the right-hand side of Eq (8) and obtain:

$$\begin{array}{c} \frac{\prod_{i}X_{i}^{s_{ij}^{+}}}{\prod_{i}X_{i}^{s_{ij}^{-}}}<{\hat{K}}_{j} \end{array}$$ (9)

where ${\hat{K}}_{j}$ are the apparent equilibrium constants defined by:

$$\begin{array}{c} {\hat{K}}_{j}=\frac{K_{j}}{\prod_{i}{{\bar{X}}_{i}}^{s_{ij}}} \end{array}$$ (10)

Under the plausible assumption that all concentrations are positive, it is allowed to use the logarithm of concentrations (concentrations are assumed to be dimensionless quantities after division by the unit concentration):

$$\begin{array}{c} y_{i}=\log X_{i} \end{array}$$ (11)

To do so, we can fix a common unit (e.g. mM) for all concentrations and equilibrium constants and neglect that unit when taking the logarithm. In the equations and inequalities, the unit would cancel anyway. Now we can write Eq (9) as (see also [37]):

$$\begin{array}{c} \sum_{i}s_{ij}y_{i}<\log{\hat{K}}_{j} \end{array}$$ (12)

In this way, we have obtained a linear inequality system. We now deal with the question whether a certain flux distribution $v\in {I\;R}^{r}$ in a metabolic system is thermodynamically feasible. This may be an elementary mode or any other flux distribution. Without loss of generality, we can assume that all fluxes are non-negative: **v**_*j*_ ≥ 0, i.e. $v\in {I\;R}_{+}^{r}$. If one flux were negative, we would simply define the orientation the other way round. Nevertheless, we can analyze various flux distributions with varying signs of fluxes. We do so consecutively for each flux distribution. Now, the inequalities Eq (12) must be fulfilled for all *j* such that **v**_*j*_ > 0, that is, for all reactions in the support *supp*(**v**) of the flux distribution **v**.

For example, consider a (hypothetical) linear chain of *n* monomolecular reactions. The chain starts with an external metabolite $M_{1}={\bar{X}}_{1}$ and ends with another one, $M_{n+1}={\bar{X}}_{2}$. Relation Eq (12) implies a chain of inequalities:

$$\begin{array}{c} \left\{\begin{array}{c} y_{2}<\log{\hat{K}}_{1}\\ y_{3}-y_{2}<\log K_{2}\\ y_{4}-y_{3}<\log K_{3}\\ \ldots \\ -y_{n}<\log{\hat{K}}_{n} \end{array}\right. \end{array}$$ (13)

Thus, each internal metabolite is confined to a certain interval in order that the flux is positive. In a large, complex metabolic network, the inequality system Eq (12) can be considered to involve many chains of inequalities. These may determine different intervals for one and the same metabolite. It is not immediately clear whether these intervals have a non-empty intersection. That is, the inequality system Eq (12) may not have any solution in *y*_*i*_ for some given *K*_*i*_ and ${\bar{X}}_{i}$. In this case, the inequality system is called inconsistent. When solving these inequalities, we can simplify them by adding inequalities, which allows us to delete all the internal logarithmic metabolite concentrations. For example, for the linear chain, this gives the following necessary consistency condition:

$$\begin{array}{c} \sum_{j=1}^{n}\log{\hat{K}}_{j}>0 \end{array}$$ (14)

We can simplify the problem enormously by considering steady-state conditions. In EFMs analysis (as in many other approaches in metabolic modeling such as FBA [43, 44] and Metabolic Control Analysis [45]), the system is assumed to be at steady state. Thus, any flux distribution *v* must fulfill the steady-state condition:

$$\begin{array}{c} \sum_{j}s_{ij}v_{j}=0\;i=1,\ldots ,m \end{array}$$ (15)

Considering all fluxes *v*_*j*_ to be non-negative (see above) is formally equivalent to assuming all reactions *j* to be irreversible. Actually, we will assume this is the case, by splitting each reversible reaction into two irreversible reactions of opposite directions. Under this condition, the set of flux distributions is a pointed convex polyhedral cone [1]:

$$\begin{array}{c} K=\{v\in {I\;R}^{r}|\sum_{j=1}^{r}s_{ij}v_{j}=0,i=1,\ldots ,m;v_{j}\geq 0\} \end{array}$$ (16)

and the EFMs are the extremal rays of K, except those extremal rays which correspond to the loops of reversible reactions that have been split.

When we check the feasibility of linear inequality system Eq (12) for a distribution flux **v**, we can use the above argument of adding inequalities. More precisely, if Eq (12) is consistent, then any positive linear combination of the inequalities which cancels the left-hand side gives a positive right-hand side. Gale’s theorem [46] (one of the Farkas-type alternative lemmas in convexity [47]) states that the reciprocal is true.

Let $\lambda \in {I\;R}^{r}$ a positive linear combination (λ_*j*_) of the inequalities Eq (12). The left-hand side gives:

$$\begin{array}{c} \sum_{j=1}^{r}\lambda_{j}\sum_{i=1}^{m}s_{ij}y_{i}=0 \end{array}$$ (17)

then λ is a solution vector in $K_{v}=K\cap \left\{v\in {I\;R}^{r}|v_{j}=0,j\notin supp\left(v\right)\right\}$.

From the right-hand side we obtain:

$$\begin{array}{c} \lambda^{T}\log\hat{K}>0 \end{array}$$ (18)

But for the vector $\log\hat{K}$ to have a positive scalar product with all vectors in $K_{v}$, it is sufficient it has a positive scalar product with all EFMs with support included in *supp*(**v**). This leads to the general result stated as Lemma 1 in [37]:

**Lemma 1** *The linear inequality system* Eq (12), *for j* ∈ *supp*(***v***), *has a solution for y if and only if the vector* $\hat{K}=\left({\hat{K}}_{1},\ldots ,{\hat{K}}_{r}\right)$ *fulfills the inequality system*

$$\begin{array}{c} e^{{\left(k\right)}^{T}}\log\hat{K}>0 \end{array}$$ (19)

*where e*^(*k*)^, *k* = 1, …, *k*_*max*_, *constitute a complete set of* EFMs *of the reaction system with support included in supp*(***v***).

Thus the linear inequality system Eq (19) is a necessary condition for **v** to be thermodynamically feasible. In particular, if the flux distribution considered is an elementary mode **e**, then the set of EFMs with support included in *supp*(**e**) is reduced to **e** itself (up to a positive scalar) and the necessary condition for thermodynamic feasibility becomes:

$$\begin{array}{c} e^{T}\log\hat{K}>0 \end{array}$$ (20)

We see that, for such an EFM, checking its thermodynamic feasibility by inequality Eq (20) is much simpler than by Eq (12), because checking consistency of an inequality system, done generally by a call to an LP program [29], is replaced by simply computing a scalar product of two vectors and checking its sign. Of course, Eq (20) requires the complete knowledge of the vector **e** with its coefficients and not only its support as in system Eq (12), but once the support is known, the coefficients are obtained easily from the nullspace of the stoichiometric matrix (limited to columns corresponding to this support), which has dimension one [48]. For a general flux distribution **v**, the complexity of using Eq (19) instead of Eq (12) depends on the number *k*_*max*_ of EFMs with support included in *supp*(**v**) and supposes these EFMs previously computed.

### 3.2 Computation of EFMs consistent with the K_*eq*_

The thermodynamic condition verifies the nice property to be monotonic with regard to set inclusion of the supports; i.e., if a given flux distribution of support **S** verifies the condition, so it is for any flux distribution with support included in **S**. This is obvious either on the form Eq (12) (a subsystem of a consistent linear inequality system is itself consistent) or the form Eq (19) (less EFMs have to be considered for a smaller support) of the condition. Thus, if a flux distribution is not thermodynamically feasible because it violates the condition, so it is for any flux distribution with a larger support.

This property is key for integrating the filtering by the condition inside the iterative process of the Motzkin Double Description (DD) method [48, 49], on which the most efficient present tools are based [14]. Recall that the DD algorithm builds in parallel, in an iterative way, the cone K and its extremal rays, thus the EFMs. In general, the nullspace approach is adopted at the initialization step, which guarantees the satisfiability of all the *m* stoichiometric equalities and of *r* − *m* irreversibility inequalities among the *r* ones defining K Eq (16). There are thus *m* iteration steps, each one integrating a new irreversibility constraint *v*_*j*_ ≥ 0 among the remaining ones. At step *l* the following cone $K_{l}$ is thus built and its EFMs computed:

$$\begin{array}{c} K_{l}=\left\{v\in {I\;R}^{r}|\sum_{j=1}^{r}s_{ij}v_{j}=0,i=1,\ldots ,m;v_{j}\geq 0,j=1,\ldots ,r-m+l\right\} \end{array}$$ (21)

Starting from the cone $K_{0}$ at initialization, the cone $K_{m}=K$ is obtained after *m* steps, as well at its EFMs. Let *supp*_*l*_(*v*) be the support of a vector $v\in K_{l}$, restricted to its first *r* − *m* + *l* components (those from which we are sure they are nonnegative). At each step *l*, 1 ≤ *l* ≤ *m*, the new cone $K_{l}$ is built by intersecting the previous one $K_{l-1}$ with the half space defined by the chosen constraint *v*_*j*_ ≥ 0 (say *j* = *r* − *m* + *l* for a convenient ordering) and its extremal rays are computed from those of $K_{l-1}$. In this process, any new extremal ray which appears is a positive linear combination of two adjacent extremal rays of the previous step. This means that any future extremal ray in the next iteration steps that a given extremal ray *e*_*l*_ at step *l* could contribute to build would have its support larger than *supp*_*l*_(*e*_*l*_). And thus, would violate the necessary thermodynamical feasibility condition if *supp*_*l*_(*e*_*l*_) does so. So, in this case, *e*_*l*_ can be definitely discarded at step *l*.

To make the filtering effective along the iteration process, it remains to check if the thermodynamic feasibility condition can be checked at each step on the restricted support of each newly created extremal ray *e*_*l*_. This can obviously be done for the condition expressed as consistency of the linear inequality system Eq (12), for all *j* ∈ *supp*_*l*_(*e*_*l*_), and achieved by a call to an LP program. This is actually what is done in ([28, 29]) with thermodynamic EFM analysis (*tEFMA*) implemented as an extension of efmtool [14] by calls to CPLEX (using actually the original form Eq (5) of inequality system, but Eq (12) could be used as well). Unfortunately, this is not the case with the simpler (without unknown variables) inequality system Eq (19). Because this condition Eq (19) involves all the complete (i.e. at final step *m*) EFMs with support included in *supp*_*l*_(*e*_*l*_), which are not yet known at current step *l*. This means we would need at step *l* the EFMs of K with support inside *supp*_*l*_(*e*_*l*_) but we only have the extremal rays of $K_{l}$. In conclusion, thermodynamical feasibility checking by inequality system Eq (19) cannot be integrated in the DD algorithm to filter out inadequate intermediate solutions during the iteration process.

### 3.3 Computations and EFMs visualization

We developed a software, thermoEFM, for performing the thermodynamic calculations explained above and a program, EFMdraw, allowing one to visualize all the EFMs. The implementation of both programs is performed with MATLAB 2015a, and they are freely available on our website https://www.lri.fr/~speres/EFM/ and figshare https://figshare.com/. They are both connected to METATOOL [12] to compute the EFMs. Celldesigner [50] was used to design the metabolic network and to visualize all the EFMs generated with EFMdraw.

## Discussion

In this work, we have compared the traditional approach in metabolic pathway analysis in which a binary distinction is made between reversible and irreversible reactions, with an approach in which more detailed thermodynamic information is used. In particular, the values of equilibrium constants and external metabolite concentrations are considered. The latter approach had been suggested even before the advent of “traditional” EFM analysis [37]. To decide which direction is realistic for a given pair of oppositely directed reversible EFMs, we have here used a Lemma described in a previous work [37]. In the present comparison, we have not, however, included the approaches in which also boundaries on internal metabolite concentrations were considered, such as [24, 28]. We have implemented the method (thermoEFM) in a MATLAB function connected to Metatool [12]. The method first computes all the EFMs and then selects those that are consistent with the formula Eq (20). This criterion is simple to be set and efficient but has to be applied in postprocessing as filtering by Eq (19) cannot be applied during the iteration steps of the Double Description algorithm. Moreover, to analyze our results, we have implemented a matlab script (EFMdraw) using Metatool and allowing the graphical representation of all the EFMs of a metabolic network designed in cellDesigner [50].

The number of EFMs on the basis of the binary declaration is often much lower than that based on the thermodynamic method. We show that in the case of a network composed of an arbitrary number of linear chains sharing one internal “hub” metabolite, the steady-state assumption allows a number of possible EFMs that is quadratic in the number of chains. Using thermodynamics in addition to the steady-state assumption divides this number by two, but it is worth to notice that knowing the direction of a number of reactions linear in the number of chains would produce the same result and even completely fix the set of EFMs. So, as the steady-state assumption already strongly constrains the directionality of reactions, only little additional gain can be expected from using thermodynamics without knowledge of the concentrations of internal metabolites, as demonstrated in Section 2.1. In particular the number of EFMs is only divided by two. Indeed, a biochemical knowledge of the irreversible directions of some reactions is enough to achieve similar or even better results than thermodynamics.

However, the advantage of our reformulation of the thermodynamic method is that formula Eq (20) only uses equilibrium constants and external metabolite concentrations which are relatively easy to obtain. And its application to biological models that consider otherwise the irreversibility of reactions, often suppresses yet some EFMs which seem to be biologically irrelevant. Moreover, for the CHO model, we have changed over time the concentrations of external metabolites as reported in [40]. The results show different capabilities of the network between culture conditions and the cell growth phases. Indeed, varying the concentrations, which are evolving during a batch culture, logically leads to different sets of tEFMs. In addition, we found three EFMs that never operate at each period of time; their infeasibility is coherent for each phase of the cell.

The question arises whether authors have sometimes declared too many reactions as irreversible. Or perhaps they were right and included, intuitively, kinetic information in addition to thermodynamics. We here suppose that some of the tEFMs are biochemically irrelevant, although they are thermodynamically feasible. This might be because they would require inhibition of some enzymes and activation of some others, i.e. regulatory mechanisms that are not taken into account. This hypothesis has been verified by several examples, e.g. monosaccharide metabolism (glycolysis and pentose phosphate pathway) [2] and a model of a network from CHO cells [40]. Taken all of the above, we argue that the binary declaration can be appropriate if done correctly and is more efficient than the thermodynamic method (but the latter, in the framework we developed with equilibrium constants, is easy to apply in addition and may suppress irrelevant EFMs).

### Conclusion

The present approach allows the testing of hypotheses on metabolic network structure as well as on the direction of fluxes. Therefore, our work contributes to expanding our knowledge on feasible metabolic networks. For reduced, incomplete networks, such as those covered in this work, results may be useful in indicating where to focus for better describing sub-networks in which only lumped reactions can be described because of lack of knowledge. In conclusion, thermodynamics is key, in the form of knowledge on reaction irreversibility or on reaction equilibrium constants, and finally experimental data on a cell behavior, enabling the reliable determination of feasible EFMs.

## Supporting information

S1 File Thermodynamical EFMs (tEFMs) of the monosaccharide metabolism. (PDF)
