# Supplementary material for: How important is thermodynamics for identifying elementary flux modes?
Source: PLoS One. 2017 Feb 21;12(2):e0171440. doi: 10.1371/journal.pone.0171440 (PMC5319754; doi:10.1371/journal.pone.0171440)
Supplement: S1 File — (PDF) [file pone.0171440.s001.pdf]

Thermodynamical EFMs (tEFMs) of the monosaccharide metabolism

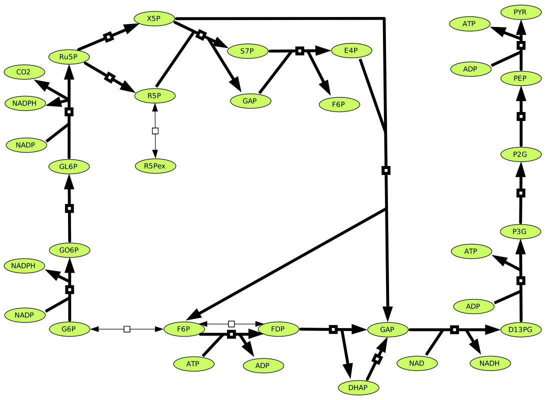

1 - tEFM 1

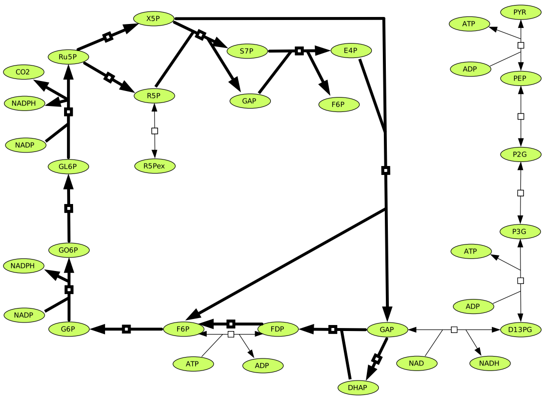

2 - tEFM 2

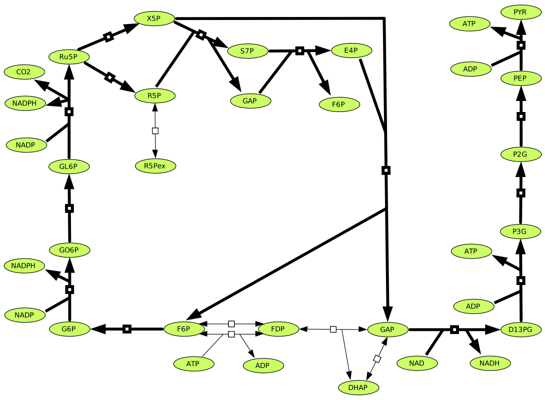

3 - tEFM 3

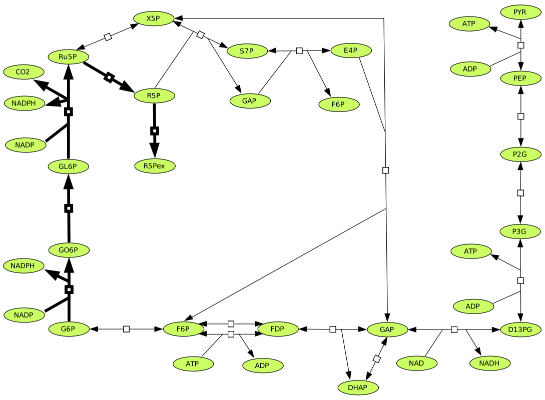

4 - tEFM 4

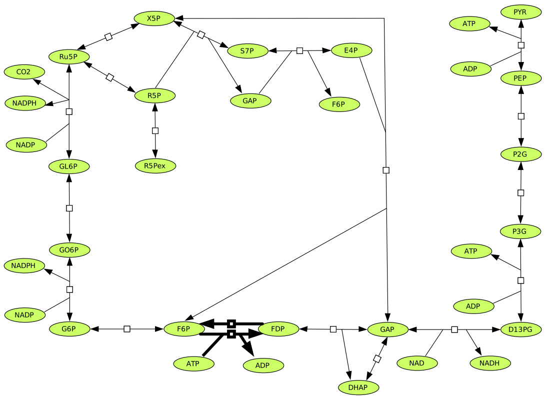

5 - tEFM 5

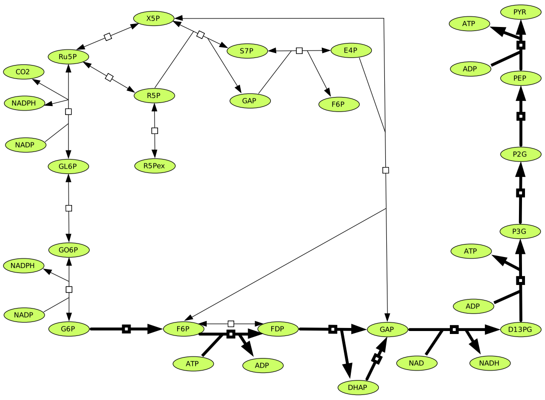

6 - tEFM 6

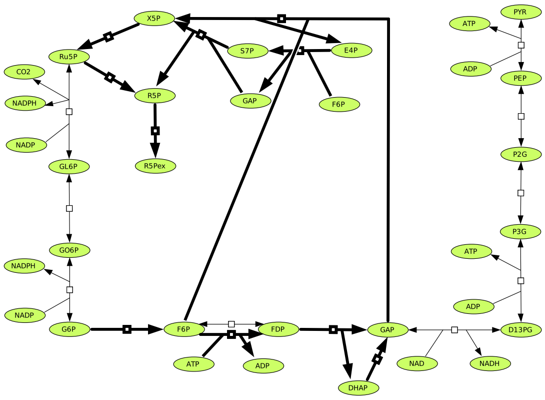

7 - tEFM 7

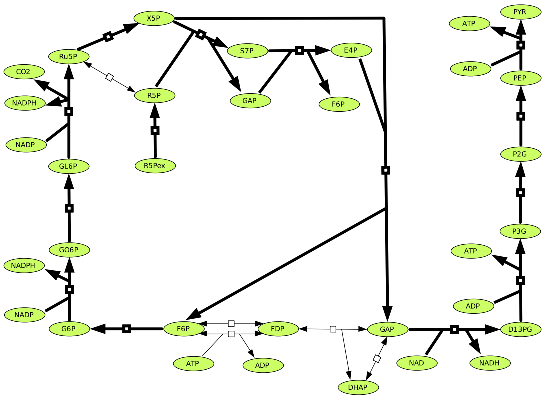

8 - tEFM 8

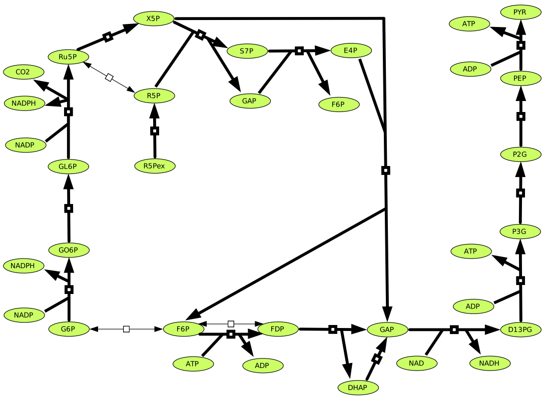

9 - tEFM 9

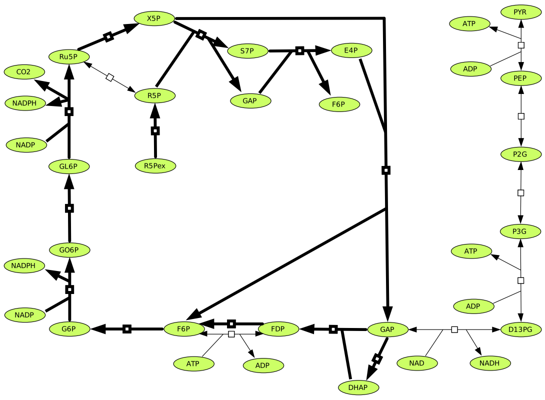

10 - tEFM 10

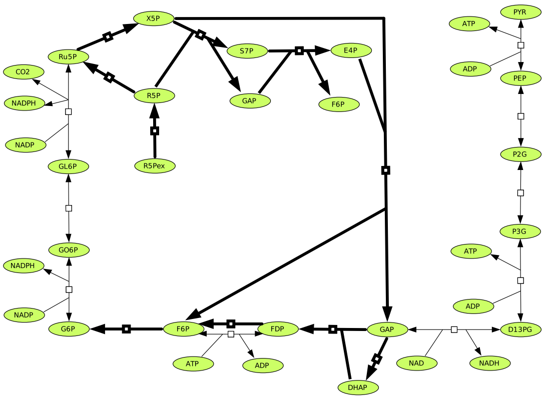

11 - tEFM 11

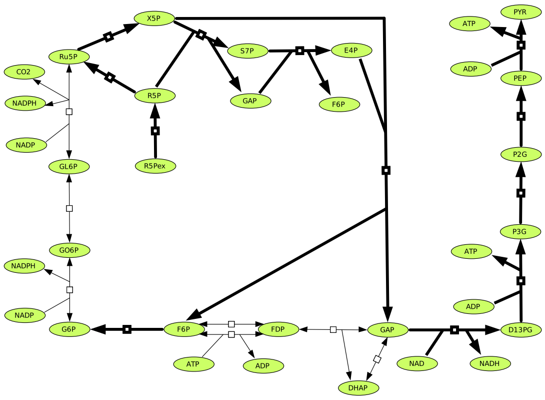

12 - tEFM 12

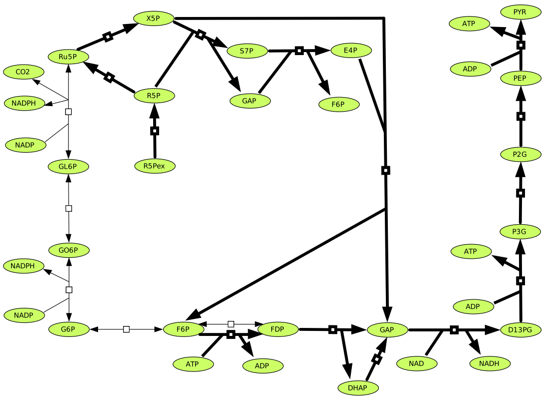

13 - tEFM 13

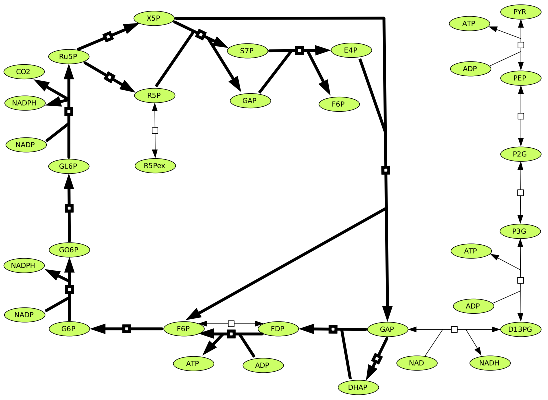

14 - tEFM 14

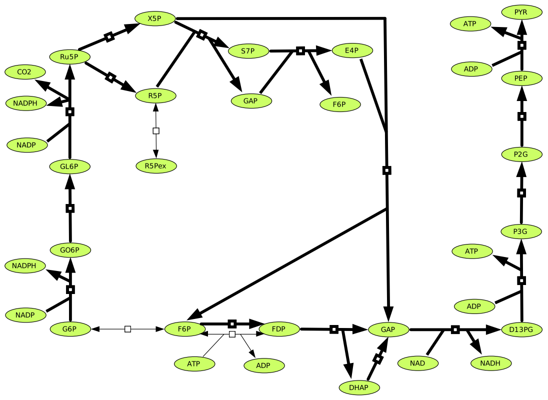

15 - tEFM 15

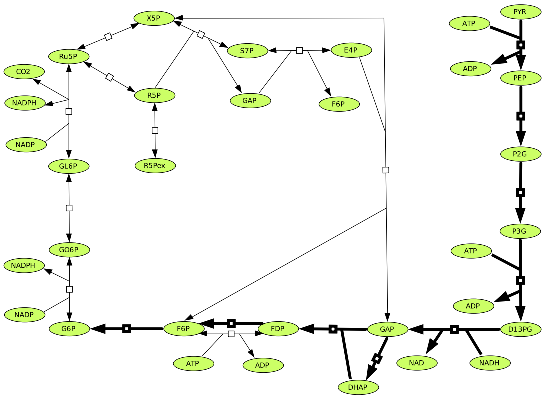

16 - tEFM 16

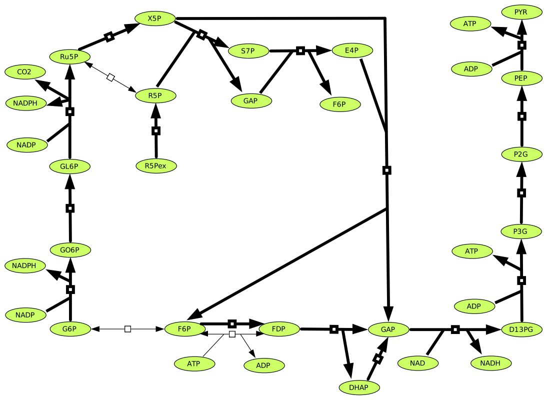

17 - tEFM 17

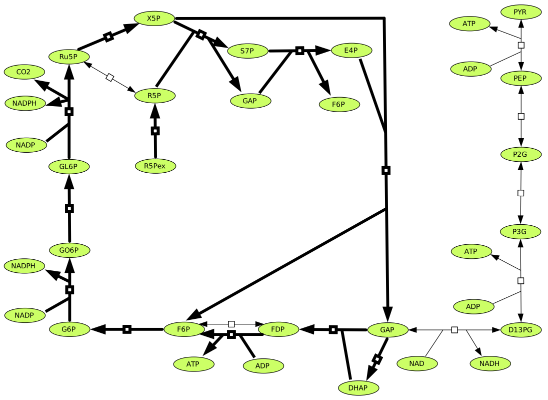

18 - tEFM 18

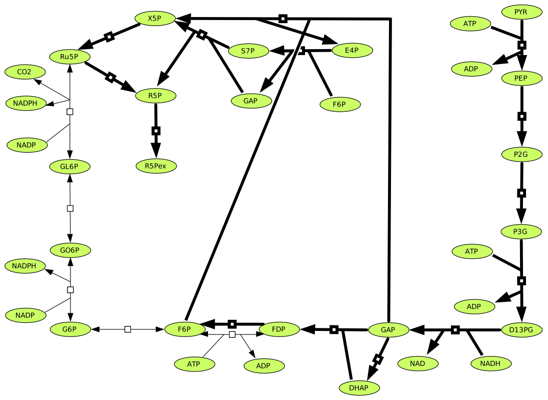

19 - tEFM 19
